# Four immunohistochemical assays to measure the PD-L1 expression in malignant pleural mesothelioma

**DOI:** 10.18632/oncotarget.25100

**Published:** 2018-04-17

**Authors:** Takuya Watanabe, Katsuhiro Okuda, Takayuki Murase, Satoru Moriyama, Hiroshi Haneda, Osamu Kawano, Keisuke Yokota, Tadashi Sakane, Risa Oda, Hiroshi Inagaki, Ryoichi Nakanishi

**Affiliations:** ^1^ Department of Oncology, Immunology and Surgery, Nagoya City University Graduate School of Medical Sciences, Mizuho-cho, Mizuho-ku, Nagoya 467-8601, Japan; ^2^ Department of Pathology and Molecular Diagnostics, Nagoya City University Graduate School of Medical Sciences, Mizuho-cho, Mizuho-ku, Nagoya 467-8601, Japan

**Keywords:** malignant pleural mesothelioma (MPM), programmed death 1 (PD-1), programmed death ligand 1 (PD-L1), immunohistochemistry (IHC), immune checkpoint inhibitors (ICIs)

## Abstract

Immune checkpoint inhibitors (ICIs) targeting the PD-1/PD-L1 pathway are expected to be a novel therapy for combating future increases in numbers of malignant pleural mesothelioma (MPM) patients. However, the PD-L1 expression, which is a predictor of the response to ICIs, is unclear in MPM. We studied the PD-L1 expression using four immunohistochemical assays (SP142, SP263, 28-8 and 22C3) in 32 MPM patients. The PD-L1 expression in tumor cells and immune cells was evaluated to clarify the rate of PD-L1 expression and the concordance among the four assays in MPM. The positivity rate of PD-L1 expression was 53.1% for SP142, 28.1% for SP263, 53.1% for 28-8, and 56.3% for 22C3. Nine cases were positive and 10 were negative for all assays. Discordance among the four assays was found in 13 cases. The concordance rates between SP142 and 22C3 and between 28-8 and 22C3 were the highest (84.4%). The concordance rates between SP263 and the other three assays were low (71.9% to 75.0%). The PD-L1 expression in MPM was almost equivalent for three of the assays. Given the cut-off values set in our study, these findings suggested that these assays, except for SP263, can be used for accurate PD-L1 immunostaining in MPM.

## INTRODUCTION

Malignant pleural mesothelioma (MPM) is a rare disease with no effective standardized systemic therapy. The median survival of untreated MPM is generally less than one year [1–3]. Multimodality therapy with surgery (extrapleural pneumonectomy or pleural decortication), chemotherapy, and radiation therapy is needed [3], but only 10%–15% of cases are completely surgically resected [2]. The standard treatment for advanced or recurrent MPM is chemotherapy with cisplatin plus pemetrexed, but the prognosis remains unsatisfactory, and the median survival is approximately only 12 months [4]. The incidence of MPM will continue to increase in Europe and Japan over the next decades due to a delay in the regulation of asbestos [1]. Therefore, it is necessary to develop more effective treatments for MPM [5].

Immunotherapies targeting the programmed death 1 (PD-1)/programmed death ligand 1 (PD-L1) pathway are a standard treatment for various malignant tumors [6–12]. Clinical trials are being conducted to investigate the effects of immune checkpoint inhibitors (ICIs) using PD-1/PD-L1 antibodies as companion diagnostic tools to determine the PD-L1 expression in MPM patients [13–15] in the same way as other malignant tumors. These immunotherapies are expected to be useful as novel therapeutic strategies to replace current therapeutic approaches [5, 16].

However, there are many PD-L1 immunohistochemistry (IHC) assays available for examining the PD-L1 expression of tumor cells (TCs) and immune cells (ICs), and selected assays vary among studies. In the Blueprint PD-L1 IHC Assay Comparison Project, the authors stained 39 cases of non-small cell lung cancer (NSCLC) using 4 PD-L1 IHC assays and reported that changing the assays and cut-off values used would lead to the misclassification of the PD-L1 status [17].

Based on these findings, we evaluated the PD-L1 expression in different histological types of MPMs using four kinds of PD-L1 IHC assays and investigated the PD-L1 expression rate in MPM and the differences in the PD-L1 expression among the four assays. The aim of this study was to establish a highly reproducible standard assessment for each companion or complementary PD-L1 antibody in MPM and to elucidate the association between the expression of PD-L1 and the clinicopathological features.

## RESULTS

### The clinical and pathological findings

The patients’ characteristics are shown in Table 1. The histological types included epithelial type (*n =* 19, 59.3%), biphasic type (*n =* 7, 21.9%), and sarcomatous type (*n =* 6, 18.8%). The study population included 27 male patients and five female patients (median age, 60.5 years; range 34–79 years). The TNM stage classifications were 1 as stage I, 4 as stage II, 17 as stage III, 8 as stage IV, and 2 as unknown because they were from biopsied cases [18]. Surgery (pleuropneumonectomy) was performed in 29 patients (90.6%); complete resection was achieved in 18 of these patients. In two patients who did not undergo surgery, tissues were obtained from thoracic biopsy specimens. One patient underwent chemotherapy, and one patient was followed with the best supportive care. The median follow-up period in all cases was 13.5 months (range, 2–117 months).

**Table 1:** Patients’ clinical data

Factor		EMM (*n* = 19)		BMM (*n* = 7)		SMM (*n* = 6)	
		Value	%	Value	%	Value	%
Sex	Male	15	78.9	6	85.7	6	100
	Female	4	21.1	1	14.3	0	0
Age (years)	median	64		57		60.5	
	range	36–72		34–77		43–79	
Stage	I	1	5.3	0	0	0	0
	II	3	15.8	1	14.3	0	0
	III	12	63.1	3	42.8	2	33.3
	IV	3	15.8	2	28.6	3	50.0
	NA	0	0	1	14.3	1	16.7
Treatment	Surgery	19	100	6	85.7	5	83.3
	Chemotherapy	0	0	1	14.3	0	0
	Best supportive care	0	0	0	0	1	16.7
Complete resection		13	68.4	3	42.9	2	33.3

Abbreviations: EMM, epithelial malignant mesothelioma; BMM, biphasic malignant mesothelioma; SMM, sarcomatous malignant mesothelioma.

### The immunohistochemical findings

**Figure 1:**
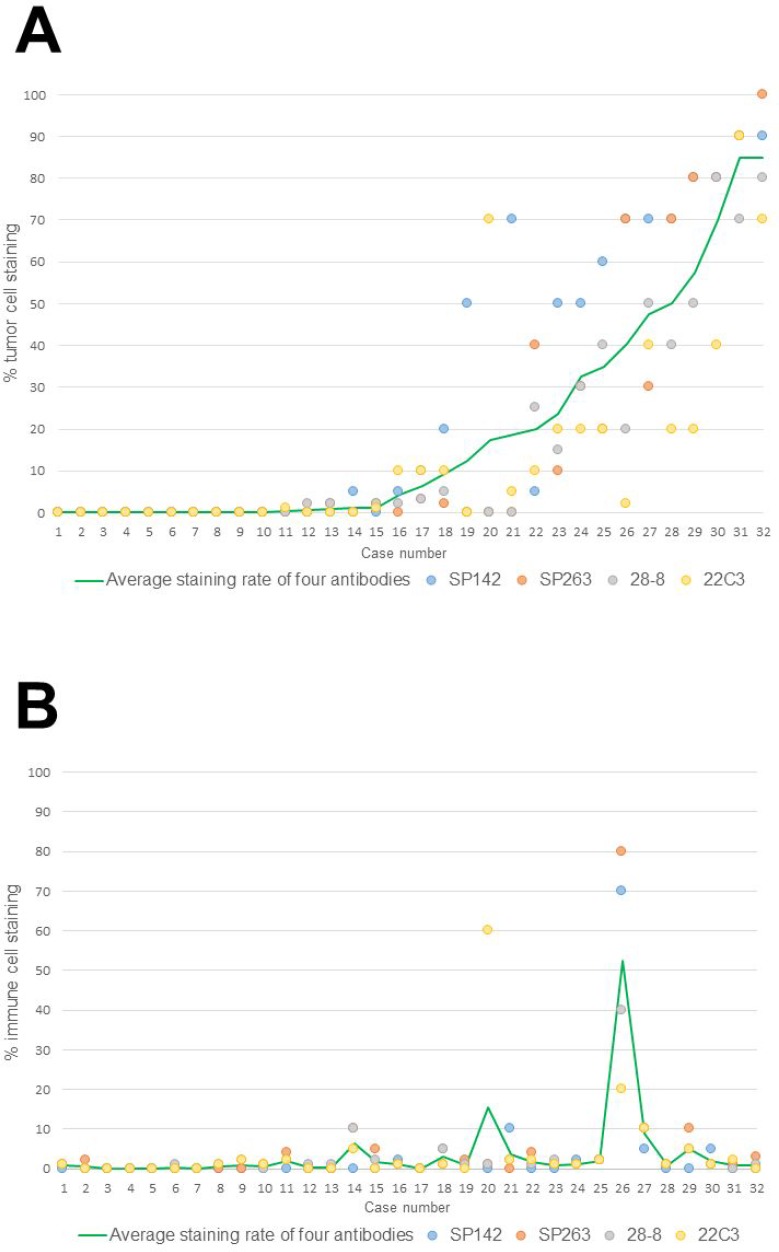
(**A**) The percentage of positivity stained tumor cells in all cases for each assay. (**B**) The percentage of positivity stained immune cells of all cases for each assay.

**Figure 2:**
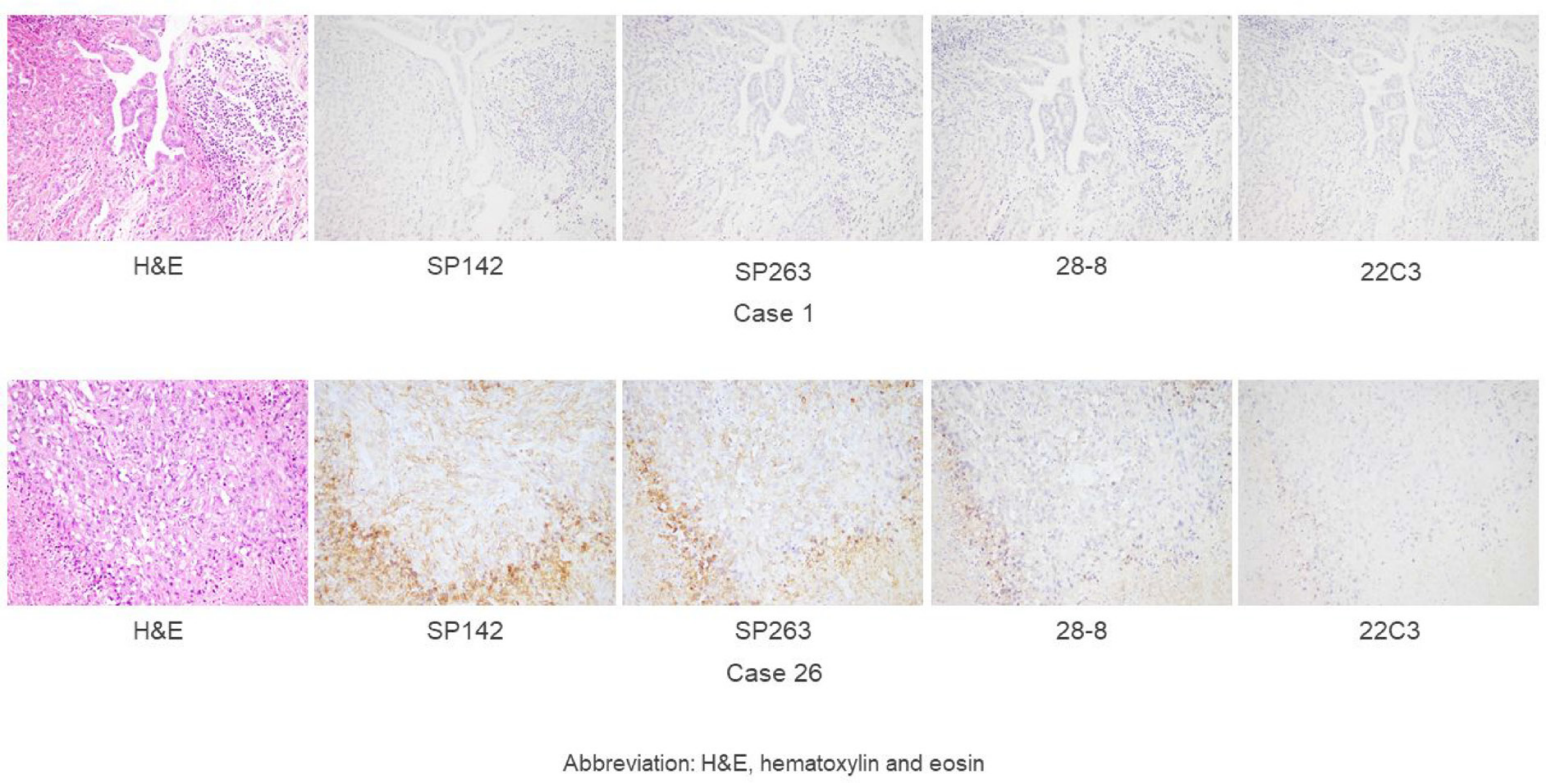
A hematoxylin and eosin-stained specimen and the representative PD-L1 expression in malignant pleural mesothelioma (magnification, ×200), as determined by the four assays (SP142, SP263, 28-8, and 22C3) Case 1 shows no staining of TCs or ICs, while Case 26 shows mid- or high- staining in TCs and ICs.

Figure 1 shows the percentages of PD-L1-positive TCs and ICs in MPM cases for each assay. The staining rate of TCs was similar in each case. Meanwhile, no correlation was found in the rates of PD-L1 staining of TCs and ICs with the four assays. As representative examples, hematoxylin and eosin (H&E)-stained and PD-L1-stained specimens (with the four antibodies) of cases 1 (negative) and 26 (positive) are shown in Figure 2.

Table 2 shows the PD-L1 expression according to the histological type of MPM, as determined by the four assays. In TCs, the rates of PD-L1 positivity were 53.1% for SP142, 28.1% for SP263, 53.1% for 28-8, and 56.3% for 22C3 (Table 2). The positivity rate of SP263 was less than that of other assays. ICs showed markedly lower rates of PD-L1 positivity in comparison to TCs.

**Table 2:** PD-L1 expression according to the histological type of MPM using the four IHC assays

Histology	SP142		SP263		28-8		22C3	
	TC	IC	TC	IC	TC	IC	TC	IC
EMM (*n* = 19)								
Positive cases	9/19 (47.4%)		3/19 (15.8%)		9/19 (47.4%)		10/19 (52.6%)	
Mean % of positivity	20.8%	1.1%	9.7%	2.8%	9.9%	2.3%	10.4%	4.8%
BMM (*n* = 7)								
Positive cases	4/7 (57.1%)		2/7 (28.6%)		4/7 (57.1%)		4/7 (57.1%)	
Mean % of positivity	28.6%	1.4%	25.0%	0.4%	22.6%	0.9%	21.4%	0.6%
SMM (*n* = 6)								
Positive cases	4/6 (66.7%)		4/6 (66.7%)		4/6		4/6	
Mean % of positivity	46.7%	12.0%	45.0%	14.3%	(66.7%)	7.5%	(66.7%)	4.2%
MPM (*n* = 32)								
Positive cases	17/32 (53.1%)		9/32 (28.1%)		17/32 (53.1%)		18/32 (56.3%)	
Mean % of positivity	27.3%	3.2%	19.7%	4.4%	16.1%	3.0%	14.3%	3.8%
Min;max of positivity	[5;90]	[1;70]	[30;100]	[0;80]	[2:80]	[0;40]	[1;90]	[0;60]

Abbreviations: MPM, malignant pleural mesothelioma; IHC, immunohistochemical; TC, tumor cell; IC, immune cell; EMM, epithelial malignant mesothelioma; BMM, biphasic malignant mesothelioma; SMM, sarcomatous malignant mesothelioma.

**Figure 3:**
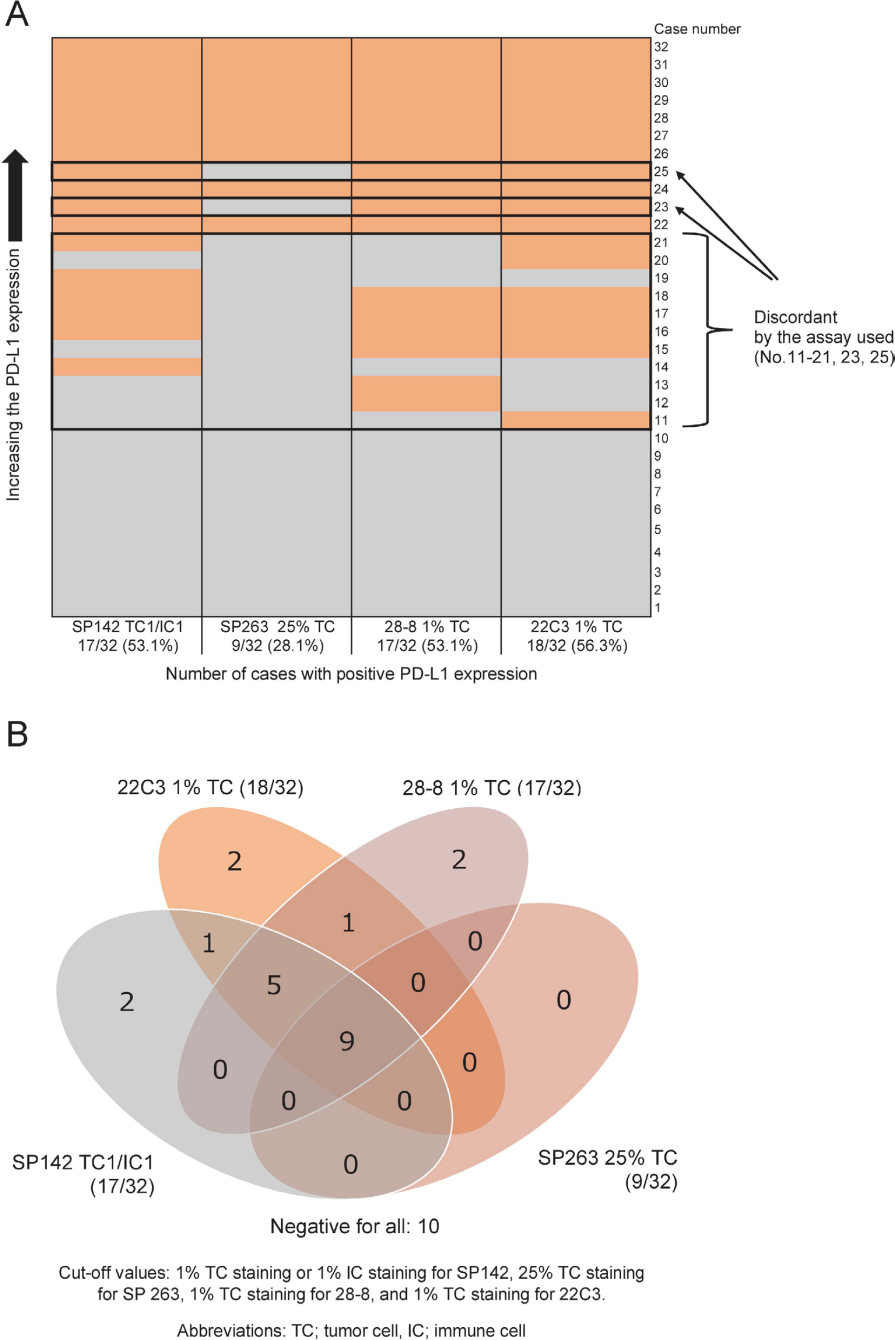
A heat map (**A**) and Venn diagram (**B**) showing a comparison of the cases using each PD-L1 IHC assay. The heat map shows the PD-L1 expression with each assay in color, with orange representing positive case and gray negative ones. The Venn diagram shows the number of cases with PD-L1 expression above each assay-specific selected cut-off value. Nine cases were positive for all assays, and 10 were negative for all assays. In 13 of 32 cases, discordance among the four assays was found (frame inset, 3A).

The heat map in Figure 3A and Venn diagram in Figure 3B illustrate, on a case-by-case basis, the cases in which TCs expressed PD-L1 for each assay. Nine cases (28.1%) were positive for all assays, and 10 (31.3%) were negative for all assays. In 13 of 32 cases (40.6%), discordance among the 4 assays was found.

Regarding the histological types, the positive rate of epithelial type was low in all assays, while the positive rate of sarcomatous type was high. In the analysis of the relationship between the PD-L1 expression and histological types (divided by epithelial type and non-epithelial type), there was no significant correlation between the PD-L1 expression and the histological type in any of the assays (SP142: *p =* 0.49, SP263: *p =* 0.06, 28–8: *p =* 0.43, 22C3: *p =* 0.62). Sarcomatous type had the highest mean percentage of positive cells in all assays except for 22C3, and epithelial type had the lowest mean percentage of positive cells in all assays.

The concordance rate between each assay is shown in Table 3. The concordance rates between SP142 and 22C3 and between 28–8 and 22C3 were the highest at 84.4%, followed by 81.3% between SP142 and 28–8. The concordance rates between SP263 and each assay were low (71.9% to 75.0%).

**Table 3:** The concordance rate between each assay

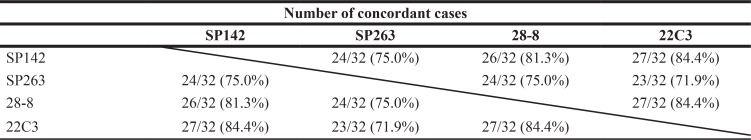

Cut-off values: 1% TC staining or 1% IC staining for SP142, 25% TC staining for SP 263, 1% TC staining for 28-8, and 1% TC staining for 22C3.

Abbreviations: TC; tumor cell, IC; immune cell.

### The analysis of the overall survival

**Figure 4:**
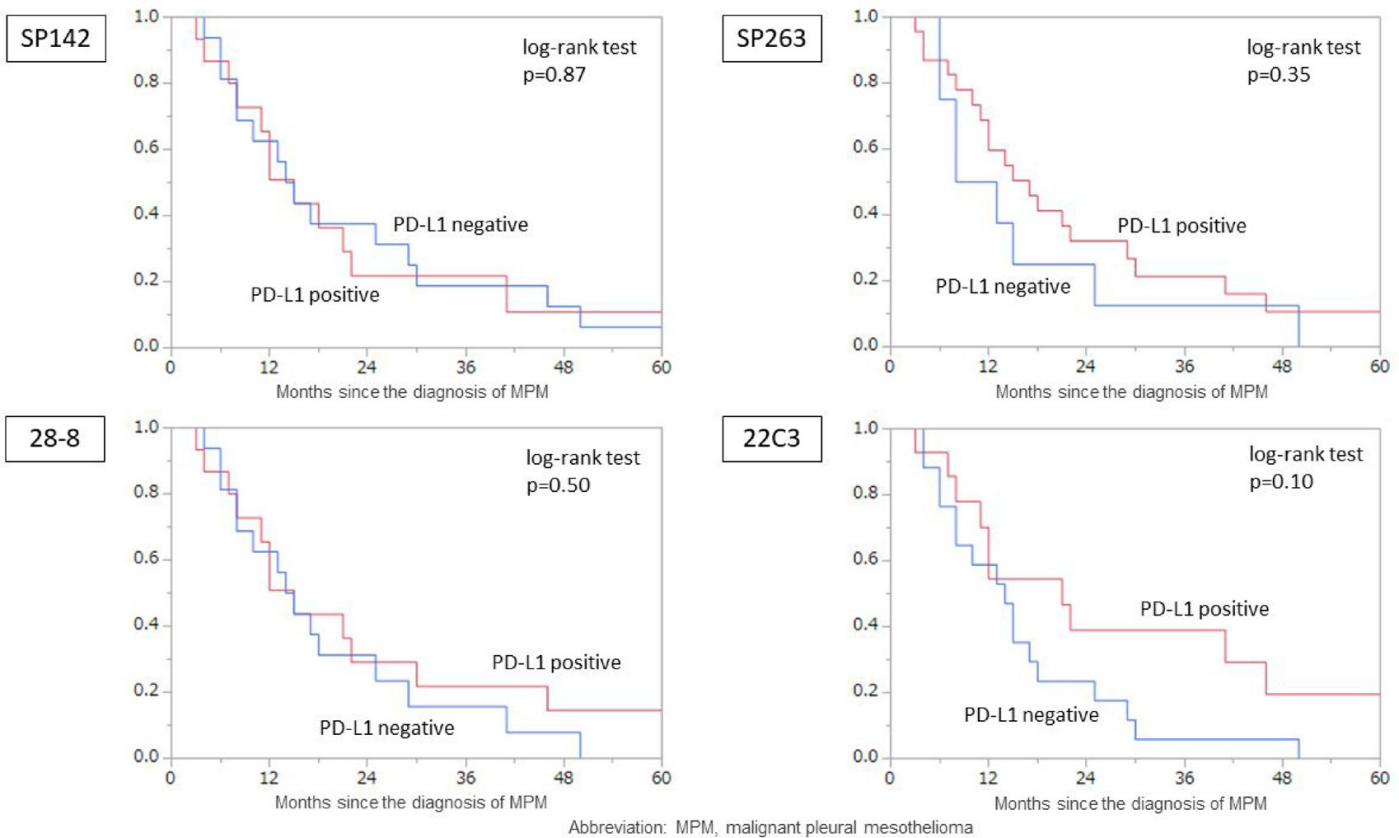
The MPM overall survival according to the PD-L1 expression of tumor cells according to each assay

In the analysis of the overall survival (OS) by the PD-L1 expression in each assay, there were no significant differences (Figure 4). We evaluated the OS in each histological type. A trend toward a poorer prognosis was noted in the PD-L1-negative cases for only 22C3, even in the analysis of epithelial type. There was no significant difference in the prognosis among all assays (SP142: *p =* 0.87, SP263: *p =* 0.35, 28-8: *p =* 0.50, 22C3: *p =* 0.10).

## DISCUSSION

MPM is a rare malignant tumor arising from mesothelial cells of the pleura and accounting for less than 0.3% of all cancers [16]. There is no doubt that asbestos exposure is responsible for the carcinogenesis of MPM. While the use of asbestos is currently prohibited, it takes many years from exposure for carcinogenesis to occur, so an increase in the number of MPM patients is expected in the coming decades [1]. The current combination therapy of surgery, chemotherapy, and radiation therapy is mostly only palliative [3]. Only 10%–15% of all MPM cases are resectable [2], and many cases recur after surgery. The standard treatment for unresectable or recurrent MPM is combination chemotherapy of cisplatin plus pemetrexed, but the median survival time of this treatment is only about 12 months, which is an insufficiently improved prognosis compared with the 10-month survival of untreated MPM [4]. A phase III randomized trial was recently conducted involving the regimen of cisplatin plus pemetrexed with bevacizumab added. The results indicated an improvement in the median OS to 18.8 months [19]. However, few patients can tolerate multidrug combination chemotherapy, so it is still necessary to establish a novel treatment strategy [5, 16].

Immunotherapies targeting the PD-1/PD-L1 pathway are now a standard treatment in various malignant tumors, such as melanoma, NSCLC, renal cell carcinoma, Hodgkin’s lymphoma, carcinoma of head and neck, and gastric carcinoma [6–12]. PD-L1, which is expressed on the surface of tumor cells and suppresses the immune response, inactivates the immune cell activity when bound to PD-1 expressed on T cells. Immunotherapy targeting the PD-1/PD-L1 pathway inhibits the binding of PD-1 to PD-L1, thereby enhancing cancer antigen-specific T cell proliferation, activation, and cytotoxic activity, bringing about an anti-tumor effect [20, 21].

Several clinical trials have been conducted to investigate the effect of immunotherapy using an ICI targeting the PD-1/PD-L1 pathway in MPM patients. The phase JAVELIN trial using avelumab (regardless of PD-L1 expression) [13], the phase IB KEYNOTE-028 study using pembrolizumab (PD-L1-positive patients) [14], and the phase II single arm NivoMes study using nivolumab [15] were held. In the JAVELIN trial, the subjects were unresectable MPM patients who experienced disease progression after chemotherapy of cisplatin and pemetrexed; these patients showed a disease control rate (DCR) of 56.6% (*n =* 53) and a median progression free survival (PFS) of 17.1 weeks [13]. In the KEYNOTE-028 study, the subjects were previously treated patients with PD-L1-positive MPM; they showed a DCR of 20% (*n =* 25), a median PFS of 5.4 months, and a median OS of 18 months [14]. In the ongoing NivoMes study, the subjects were progressive MPM patients; a DCR of 50% (*n =* 34) was reported at the 2016 World Conference on Lung Cancer [15]. Based on these findings, ICIs are expected to be useful as a novel treatment strategy for MPM that replaces or adds to the current systemic treatment [5, 16].

Many PD-L1 IHC assays have been developed to examine the PD-L1 expression of TCs, and the assays used vary among studies and ICIs. In the Blueprint PD-L1 IHC Assay Comparison Project, the authors stained 39 cases of NSCLC using four PD-L1 IHC assays. They reported that changing the assays and cut-off values used would lead to the misclassification of PD-L1 status [17]. Given the large number of PD-L1 IHC assays and cut-off values for positivity for PD-L1 expression, selecting patients who are adaptive to immunotherapies for malignant tumors, including MPM, is difficult [16]. We therefore evaluated the PD-L1 expression of different histological types of MPM using four PD-L1 assays that are also diagnostic for NSCLC and investigated the differences in the PD-L1 expression among the assays.

The PD-L1-positive rate of MPM differs wildly among reports, ranging from around 20% to about 70% [22–24]. In the NivoMes study, the positive rate of PD-L1 expression was 28% using a cut-off of ≥1% TC staining for 28-8 [15]; in the JAVELIN trial, the positive rate was 35.9% using a cut-off of ≥5% TC staining [13]. Thapa *et al.* and Mansfield *et al.* reported values of 41.7% and 40% with the same cut-off [25, 26]. Given that these reports used different assays and cut-off values, it seems obvious that their results would differ as well. In the present study, the positive rate of PD-L1 expression differed depending on the assay: 53.1% for SP142, 28.1% for SP263, 53.1% for 28-8, and 56.3% for 22C3. This result is considered to depend not only on the difference in assays but also on how the positive cut-off for the PD-L1 expression is determined.

The cut-off values in the present study were the same as those used in the Blueprint project [17]. SP263 is a companion diagnostic assay of durvalumab, and PD-L1 expression was set as positive for this assay with a cut-off of 25% TC staining [19]. An examination of the clinicopathological features of five cases that were PD-L1-negative only in SP263 assay showed that three were epithelial type, two were biphasic type, and all had undergone surgical resection. Therefore, there were no remarkable clinicopathological features. However, the proportion of positive cells with the other 3 assays in these 5 cases was mostly <25%. When the cut-off value of SP263 was set at 1% TC, as in the other 3 assays, 4 of 5 cases were evaluated as positive. The total positivity rate of PD-L1 expression was as high as 46.9% (epithelial type 7/19, biphasic type 4/7, sarcomatous type 4/6), showing almost the same positivity rate as the other 3 assays. Based on these findings, given that a relatively high staining ratio was required for this assay compared with others, few positive cases were detected.

Regarding the PD-L1 expression by histological types of MPM, some reports have found that PD-L1-positive cases were more frequently non-epithelial type (especially sarcomatous type) than epithelial type [22, 23, 25], but no correlation was found in the PD-L1 expression between epithelial and non-epithelial type in any of the assays in this study. In addition, some reports of MPM have shown that PD-L1-positive cases have a poorer prognosis than negative cases [22, 23, 26], but no correlation was found in the prognosis between PD-L1-positive cases and PD-L1-negative cases in this study. This is probably due to the small number of cases examined. More cases should be accumulated, and the findings revised. In malignant tumors other than MPM, contrasting results have been reported regarding the relationship between the PD-L1 expression and the prognosis. For example, the PD-L1 expression was found to be a good prognosis factor in NSCLC, colorectal cancer, and thymic carcinoma [27–29], but a poor prognosis factor in lung squamous cell carcinoma, renal cell carcinoma, and gastric carcinoma [30–32].

Among the four assays used in this study, SP142 is reported to be less likely to stain TCs in NSCLC or MPM than the other three assays [17, 23, 33]. However, the SP142 staining was equivalent to 28-8 and 22C3, and the percentage of positive cells with SP142 was highest among the four assays used in this study. A number of reasons may explain for this discrepancy. First, the conditions of the collected samples differ according to each study. In addition to the patient treatment history, the histological type, processing, storage, and amount of tumor tissue might affect the ability to detect PD-L1 in each case [29]. It has been reported that surgical specimens show a higher rate of PD-L1 positivity than biopsy specimens [34, 35]. In addition, previous reports have shown that the PD-L1 expression is heterogeneous at different sites within the same specimen [36], and that it differs according to the effects of exposure to radiotherapy or chemotherapy [35]. The small number of samples was a major limitation of the present study. However, most of the samples (93.8%) were surgical specimens, and none of the samples were from patients who had been exposed to radiotherapy or chemotherapy. If we had included more biopsy specimens after treatment in our study, we may have obtained different results for the PD-L1 expression. Second, SP142 and SP263 antibodies bind to the intracellular domain of PD-L1, while 22C3 and 28-8 antibodies bind to the extracellular domain [16]. This difference in the binding domains alters the sensitivity and specificity of the detection assay. In MPM, this difference may have a greater effect on the results than it does in other carcinomas. Furthermore, Yu *et al.* reported that slides stained within 90 days had a slightly higher prevalence of PD-L1 positivity in comparison to specimens that were stored for ≥90 days [37]. The denaturant effect of formalin fixation on protein could also compromise antigen staining during immunohistochemistry. In this study, all specimens were stained immediately after slicing. In addition, all specimens were also stained with the IHC antibodies specific for MPM, such as calretinin, cytokeratin 5/6, and vimentin [38, 39], to confirm that tumor antigenicity was completely maintained. Actually, 19 of 32 samples were obtained more than five years previously. However, no correlation was found between the old samples and the new samples with regard to the rates of PD-L1 positivity among any of the assays. The current use of such non-standardized immunohistochemical techniques to measure the PD-L1 expression in tissue might have some effect on the results. Of course, it should be noted that the results of this validation trial for MPM are not necessarily similar to the results for NSCLC. At any rate, it will be important to develop standardized methods for evaluating the PD-L1 expression by immunohistochemistry.

In IC staining, no significant correlations were observed among the four assays. Tumor-infiltrating lymphocytes (TILs) are measured morphologically, and there is currently no established threshold for TILs [40]. However, the presence of TILs—a key component of the tumor microenvironment—is a good prognostic factor in numerous cancers [41–43]. In the future, it will be necessary to establish a more objective and simple method for evaluating IC staining, and the significance of the PD-L1 expression in ICs should be analyzed.

To the best of our knowledge, this is the first validation trial for MPM using the four companion diagnostic assays. In addition, most of the samples were surgical specimens, and the results from quality-controlled samples were verified by staining with IHC antibodies specific for MPM. We believe that this research gives MPM patients very realistic information that will be useful in the clinical administration of ICIs.

Finally, as a future issue of immunostaining for MPM, it is important to determine whether or not the four PD-L1 IHC assays have specific staining properties for any TCs, as some MPMs have a heterogeneous tissue morphology within the tumor [44–46]. Therefore, the nine cases found to be PD-L1-positive in all assays in this study should have their staining distribution checked for each assay on a cell-by-cell basis.

In conclusion, the positive rate of PD-L1 expression in MPM was over 50% and almost equivalent among the SP142, 28-8, and 22C3 assays. The relatively low rate of PD-L1-positive cases with the SP263 assay may have been due to the cut-off value used. Our results suggest that ICIs might be effective for MPM as novel therapeutic agents, and the three assays with the cut-off values used in our study may all be suitable for accurate PD-L1 immunostaining in MPM.

## MATERIALS AND METHODS

### Patients and specimens

Thirty-two MPM tissue specimens were obtained by surgical excision (including two thoracoscopic biopsy specimens) from January 1992 to December 2016 at Nagoya City University Hospital. All cases were microscopically reviewed and diagnosed by two expert pathologists (TM and HI). All specimens were stained with IHC antibodies specific for MPM such as calretinin, cytokeratin 5/6, and vimentin [38, 39], and it was confirmed that tumor antigenicity was completely maintained. No specimens were obtained from patients who had undergone preoperative radiotherapy or chemotherapy, and no specimens associated with any therapeutic trial or ICI therapy were included in this study. The relevant clinical data were collected from medical records. This study was approved by the Institutional Review Boards of Nagoya City University Hospital and carried out in accordance with the Declaration of Helsinki. All of the patients consented to the use of their tissues for the present analysis.

### PD-L1 immunohistochemistry

An appropriate formalin-fixed and paraffin-embedded (FFPE) block containing the tumor in each case was selected by reviewing H&E-stained specimens, and each FFPE block was sliced into 3-μm-thick tissue sections. The tissue sections were deparaffinized and subjected to immunostaining with the following for anti-PD-L1 antibodies: clone SP142 (Ventana Medical Systems, Tucson, AZ, USA), clone SP263 (Ventana Medical Systems), clone 28-8 (Dako, Carpentaria, CA, USA), and clone 22C3 (Dako). SP142 and SP263 immunostaining was carried out with a Bond-Max autoimmunostainer (Leica Microsystems, Wetzlar, Germany) and a Bond polymer-refine detection kit (Leica Microsystems). 28-8 and 22C3 immunostaining was carried out with a Dako autostainer Link48 system (Dako) and a PD-L1 PharmDx kit (Dako).

### PD-L1 scoring

The positivity of TCs and ICs was assessed by two expert pathologists (TM and HI). TCs in which the membrane was immunostained at any intensity were considered to be positive for PD-L1. The ratios of PD-L1-positive TCs were evaluated by microscopic observation. Meanwhile, ICs in which the membrane or cytoplasm was immunostained at any intensity were considered to be positive for PD-L1 because the stained membrane and cytoplasm could not be distinguished in lymphocytes due to their small size [29]. Most PD-L1-positive ICs were macrophages and lymphocytes. ICs were quantified by evaluating the ratio of the area covered by stained ICs in the tumor area, as described in previous reports [47–49]. The tumor area was defined as the area occupied by viable TCs and their associated intratumoral and contiguous peritumoral stroma [41]. The necrotic areas were excluded from the scoring area. Although cases with <100 viable TCs were excluded from the present study; all examined cases contained >100 TCs. Negative reagent controls were evaluated in each case by confirming the acceptable level of background staining. The cut-off values were set at 1% TC or 1% IC for SP142, 25% TC for SP263, 1% TC for 28-8, and 1% TC for 22C. The cut-off positive staining ratio was determined based on the clinical response to anti PD-1/PD-L1 therapy in previous reports [7–9, 47–51].

### Statistical analysis

The analysis was performed using a Fisher’s exact test for qualitative variables. Survival curves were generated using the Kaplan-Meier method, and the log-rank test was used to assess the statistical significance of the differences between groups. The two-sided significance level was at *p* value < 0.05. We performed all analyses using the JMP software program (version 12.0.1, SAS Institute, Tokyo, Japan).

## Abbreviations

MPM: malignant pleural mesothelioma
PD-1: programmed death 1
PD-L1: programmed death ligand 1
ICI: immune checkpoint inhibitor
IHC: immunohistochemistry
NSCLC: non-small cell lung cancer
TC: tumor cell
IC: immune cell
OS: overall survival
DCR: disease control rate
PFS: progression free survival
TILs: tumor-infiltrating lymphocytes
FFPE: formalin-fixed and paraffin-embedded
H&E: hematoxylin and eosin
